# Peto's paradox revisited: theoretical evolutionary dynamics of cancer in wild populations

**DOI:** 10.1111/eva.12025

**Published:** 2012-11-22

**Authors:** Benjamin Roche, Kathleen Sprouffske, Hassan Hbid, Dorothée Missé, Frédéric Thomas

**Affiliations:** 1 IRD, UMMISCO (UMI IRD/UPMC)Bondy, France; 2 CREEC, Université Montpellier 2Montpellier, France; 3 Institute of Evolutionary Biology and Environmental Studies, University of ZurichZurich, Switzerland; 4 Laboratoire de Mathématiques et Dynamique de Populations, Cadi Ayyad UniversityMarrakech, Morocco; 5 IRD, MIVEGEC (UMR CNRS/IRD/UM1)Montpellier, France

**Keywords:** biomedicine, disease biology, evolutionary medicine, evolutionary theory

## Abstract

If the occurrence of cancer is the result of a random lottery among cells, then body mass, a surrogate for cells number, should predict cancer incidence. Despite some support in humans, this assertion does not hold over the range of different natural animal species where cancer incidence is known. Explaining the so-called ‘Peto's paradox' is likely to increase our understanding of how cancer defense mechanisms are shaped by natural selection. Here, we study how body mass may affect the evolutionary dynamics of tumor suppressor gene (TSG) inactivation and oncogene activation in natural animal species. We show that the rate of TSG inactivation should evolve to lower values along a gradient of body mass in a nonlinear manner, having a threshold beyond which benefits to adaptive traits cannot overcome their costs. We also show that oncogenes may be frequently activated within populations of large organisms. We then propose experimental settings that can be employed to identify protection mechanisms against cancer. We finally highlight fundamental species traits that natural selection should favor against carcinogenesis. We conclude on the necessity of comparing genomes between populations of a single species or genomes between species to better understand how evolution has molded protective mechanisms against cancer development and associated mortality.

## Introduction

In the transition to multicellularity (Szathmáry and Smith 1995), cells within the body have evolved to cooperate with each other. However, cheating cells can emerge and invade nearby cell populations, sometimes leading to cancer (Cairns 1975). The carcinogenic process is largely shaped by the alteration in gene expression, through genetic or epigenetic mutations (Stratton 2011) that lead to the deregulation of genes controlling the cell cycle (Hanahan and Weinberg 2000, 2011). Furthermore, these alterations can be either inherited, leading to an increased susceptibility to cancer (Knudson 1971), or acquired in somatic cells, the probability of which is modulated by environmental factors and mutagen exposure such as tobacco smoke (Doll and Peto 1981). Understanding how evolution has selected for protective mechanisms against deregulation of key cellular functions that characterize cancers is of primary interest in the struggle against this potentially lethal disease (Greaves and Maley 2012).

It is currently thought that carcinogenesis involves two main classes of genes: proto-oncogenes and tumor suppressors. Mutations in proto-oncogenes are like ‘stuck accelerators’ in cars, and mutations in tumor suppressor gene (TSGs) are like ‘dysfunctional brakes’ (Vogelstein and Kinzler 2004). Proto-oncogenes are generally dominant so that activation only requires one mutation (then the proto-oncogene becomes an oncogene), while TSGs are generally assumed to be recessive and thus require two mutations to be inactivated (Knudson 1971, 2001, but see Berger et al. 2011. Proto-oncogenes, such as genes regulating cellular proliferation, increase the probability of cancer when activated (Vogelstein and Kinzler 2004). Of note, carcinogenesis generally requires that mechanisms of DNA repair, such as TSGs, be inactivated (Knudson 1971; Berger et al. 2011).

If the probability of oncogene activation and TSG inactivation is identical at each somatic cell division across different species (e.g., the same number of copies), then all else being equal, individuals from large body size taxa, such as whales, should be more prone to cancers than relatively smaller organisms, such as mice, simply because they have more cells. While this scaling between number of cells and cancer frequency seems partially true at an intraspecies level, for example, in humans (Albanes and Winick 1988; Albanes 1998; Thomas et al. 2012), empirical evidence does not support this prediction in natural animal populations, in which cancer frequency typically ranges from 20% to 40% across all sampled species and is not related to body mass (Peto et al. 1975; Caulin and Maley 2011). This discrepancy between data and theory is the so-called ‘Peto's paradox’, based on a study showing that cancer in mice is dependent on mutagen exposure rather than age (Peto et al. 1975). Resolving this paradox is a promising research avenue for understanding how some natural animal species have evolved particular mechanisms conferring added protection against cancer.

Different mechanisms can constrain oncogene activation or TSG inactivation. One fundamental mechanism is simply the number of copies of each gene. For instance, redundancy of TSGs could buffer their inactivation by requiring more mutations (Nunney 1999; Leroi et al. 2003; Seluanov et al. 2008; Caulin and Maley 2011). Despite works discussing the possible mechanisms involved (Nunney 1999; Caulin and Maley 2011), we know little about the selective forces at play in the stability of proto-oncogenes and TSGs in natural animal population. It has been recently suggested that mechanisms leading to cancer avoidance should be under increasing positive selection along a body mass gradient, because the risk of cancer and associated mortality should increase in a similar manner (Caulin and Maley 2011; Roche et al. 2012).

Here, we study the effect of body mass on the evolutionary dynamics of TSG inactivation and oncogene activation across a broad range of masses, ranging from 10 gram to 1 ton (as a reference, mice weigh 20 grams on average, humans 70 kg, and whales 20 tons). Using a mathematical model, we show that the rates of TSG inactivation and oncogene activation should be nonlinear along a gradient of body mass. More specifically, we identify a threshold in body mass beyond which the benefits of reduced TSG inactivation rates do not overcome their costs, leading to an equilibrium population that differs from the expected carrying capacity. We also show that oncogenes may be frequently activated first within populations of large organisms, suggesting that TSG inactivation is more frequently the second step to cellular deregulation and acts as the final trigger of tumor emergence. We then discuss experimental settings that can be employed to identify protective mechanisms against cancer. Finally, we highlight the fundamental species traits that evolution should favor against tumorigenesis. We conclude by discussing the necessity of comparing genomes between populations of a single species or those between species if we are to better understand how evolution has molded protective mechanisms against cancer development and associated mortality.

## Materials and methods

To date, theoretical studies addressing the evolutionary dynamics of TSG inactivation or oncogene activation in humans have mainly focused on within-host scales (Nunney 1999; Nowak et al. 2004; Nagy et al. 2007).

Here, we adopt a complementary approach by considering a similar cancer type occurring in different natural animal species. We base our within-host model on previous work (Nowak et al. 2004) and add to this mathematical terms of population-scale selection, as detailed below.

### Intra-organism model

We modeled the probabilities of oncogene activation and TSG inactivation as a function of the number of cells within an individual. Oncogene activation is generally a dominant, genetically determined character (Vogelstein and Kinzler 2004), requiring only a single mutation (quantified here as *u*_0_). Thus, the probability *P*(*t*) that a single cell has an oncogene activated by one hit at time *t* can be expressed through the cumulative distribution function of the exponential law:


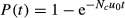


where *N*_*c*_ is the number of cells and *u*_0_ is the mutation rate for oncogene activation. We assume that the activation rate is the inverse of the time until the probability of activation reaches 50%, leading to the following rate of oncogene activation *σ*_*O*_:


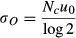


Following Knudson's hypothesis (Knudson 1971, 2001), we assume that TSGs are recessive and two mutations are required to inactivate their expression (characterized by *u*_1_ and *u*_2_). As previously shown by Nowak et al. (2004), TSG inactivation can be modeled as a branching process with two consecutive steps. Assuming that cell population *N*_*c*_ is sufficiently large, the probability that a single cell has a TSG inactivated by two hits at time *t* can be expressed as follows:


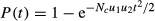


where *N*_*C*_ is the cell population size and *u*_1_ and *u*_2_ are the mutation rates for the first and second events, respectively.

As before, we assume that the rate of TSG inactivation is characterized by the inverse of the time until probability of inactivation is 50%. Using the derivation obtained by Nowak et al. (2004), the rate of TSG inactivation is as follows:


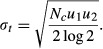


Finally, we assume that the rate of TSG inactivation includes both the emergence of cells with inactivated TSGs as well as their fixation in cell population, even if each mutation does not lead necessarily to fixation (Durrett and Schmidt 2008).

### Evolutionary strategies: benefits and costs

Oncogene activation and TSG inactivation depend on the number of cells *N*_*c*_ and mutation rates, respectively (*u*_0_, *u*_1_, *u*_2_). Assuming that *u*_2_ is a constant, mutation rates *u*_0_ and *u*_1_ are the evolving strategies of the species within this framework.

**Figure 1:**
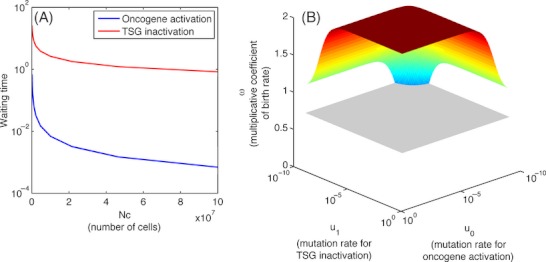
(Left) Waiting time for oncogene activation (blue) and tumor suppressor gene (TSG) inactivation (red) as a function of the number of cells in the organismwhich is surrogated by body mass species. The underlying branching process is described in the main text. (Right) Trade-off between rates of oncogene activation (*u*_0_), first hit of TSG inactivation (*u*_1_), and the corresponding multiplicative factor of birth rate (ω). Gray plane represents the death rate scaled to the allometric birth rate (where *z*-axis equals to 1). Trade-off is explained in the main text. Parameters used are as follows: (Left) *u*_0_ = 10^−5^, *u*_1_ = 10^−5^, *u*_2_ = 10^−3^. (Right) *c* = 0.5, *g* = 2 × 10^−9^.

Mutation rates in our model reflect the rate of change to any activated state within a cell. If we assume that the somatic mutation rate is similar across species as it has been previously suggested for mice and humans (Trosko and Chu 1975; but see Turker 1998), then *u*_0_ and *u*_1_ are implicitly equal to the number of proto-oncogenes and TSGs, respectively, multiplied by the somatic mutation rate. In other words, if protective mechanisms are mainly the number of copies of TSGs and proto-oncogenes (but see Humbert et al. 1999; Savage et al. 2007 for other possibilities), then *u*_0_ and *u*_1_ will be lower if protection mechanisms are present and greater if absent. Thus, we do not explore the relationship between body mass and mutation rate, but rather the relationship of body mass with the number of proto-oncogenes and TSGs contained in the genome. Lower values of *u*_0_ and *u*_1_ in our model are equivalent to increasing the number of copies of proto-oncogenes and TSGs, respectively, which decreases the probability that oncogenes are activated and TSGs are inactivated. Mutations in both oncogenes and TSGs lead to carcinogenesis, and here, we assume that the mechanisms to reduce mutations have a cost for the organism in terms of decreased birth rate (Moses and Brown 2003). To account for this in our model, the birth rate is then multiplied by a coefficient *ω*, which formalizes the trade-off between *u*_0_, *u*_1_, *u*_2_, and birth rate. Thus, there is no protection against cancer with high mutation rates (*u*_0_ and *u*_1_) and good protection with low. This allows us to simulate the cost of these protections. In mathematical terms, we assume a saturating and symmetric trade-off involving *u*_0_, *u*_1_*,* and *ω* (Fig. 1):


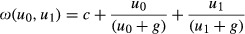


where *c* and *g* are two constants affecting the shape of the trade-off. Then sensitivity of the results to changes in these parameters is analyzed in the Supporting information.

### Interorganism model

We embed the previously developed intra-organism model into a population-scale model. We assume that the population is subdivided into distinct genotypes as follows:


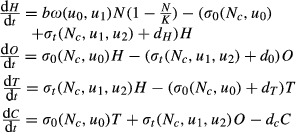


where *N* is the total population size. Healthy individuals (*H*) give birth at a rate *b* that is multiplied by the function *ω*(*N*_*c*_,*u*_0_,*u*_1_,*u*_2_), which, as earlier described, represents the trade-off between protective mechanisms and number of cells on reproductive fitness. We characterize intrapopulation competition using a logistic function with carrying capacity *K*, as is standard practice for modeling populations (Sibly et al. 2005). Healthy individuals can become oncogene activated (*O*) through mutation with rate σ_*O*_ or TSG inactivated (*T*) with rate σ_*T*_; either genotype *O* or *T* can acquire a second mutation and harbor both phenotypic changes *O* and *T*. We assume that this last state ‘cancer’ (C) represents individuals who have an increased probability of death resulting from both activation of oncogenes and inactivation of TSGs. Our model does not incorporate additional mutations that may be necessary for some types of cancer (Vogelstein and Kinzler 2004). Mortality rates (*d*) may differ among the four phenotypic states.

### Numerical simulations

For simplicity, we have only described the model with a single strategy, that is, described by a single combination of *u*_0_ and *u*_1_. Strategy evolution requires that we extend the model through a mutation parameter *m* that permits different combinations of *u*_0_ and *u*_1_ (see Supporting information). Ten different values for each of *u*_0_ and *u*_1_, ranging from 10^−10^ to 10^−1^, are explored through simulations, leading to 100 possible strategies. We employed numerical simulations to determine the prevalence of each strategy (represented by a pair of *u*_0_ and *u*_1_) within the population after 4000 generations of simulated evolution. Initial population sizes *N* were uniformly distributed between these different strategies.

Because we are interested in the effects of body mass (*M*) on cancer evolutionary dynamics, we employed previously described allometric laws (De Leo and Dobson 1996; West et al. 1997) to obtain plausible parameters for our simulations (Table 1). We also assumed that *d*_*H*_ = *d*_*O*_ = *d*_*T*_ = *d* and *d*_*C*_ = ρ*d*, where ρ is the coefficient of cancer mortality, to address the costs exerted by cancer on organism fitness (this assumption is relaxed later, when potential experiments are addressed).

**Table 1:** Parameters of the model and their associated allometric relationships. See text for further explanation

Notation	Meaning	Relationship	Reference
b	Maximum population birth rate	b = 0.6 *M*^−0.27^	De Leo and Dobson (1996)
d	Intrinsic population death rate	d = 0.4 *M*^−0.26^	De Leo and Dobson (1996)
K	Population carrying capacity	K = 16.2 *M*^−0.7^	De Leo and Dobson (1996)
*N*_c_	Number of cells in each individual	*N*_*c*_ = *M*	
*ρ*	Multiplicative coefficient of natural mortality for individuals with both oncogene activated and TSG inactivated	10	
ω	Multiplicative coefficient of the birth rate *b* used to simulate cost of cancer protection mechanisms	[0.5–2]	
*c,g*	Constants for c = 0.5, g = 2 9 10^∧-9^the trade-off shape		
*m*	Mutation rate between evolutionary strategies	0.002	

TSG, tumor suppressor gene.

## Results

### Influence of body mass on the evolution of protection mechanisms

**Figure 2:**
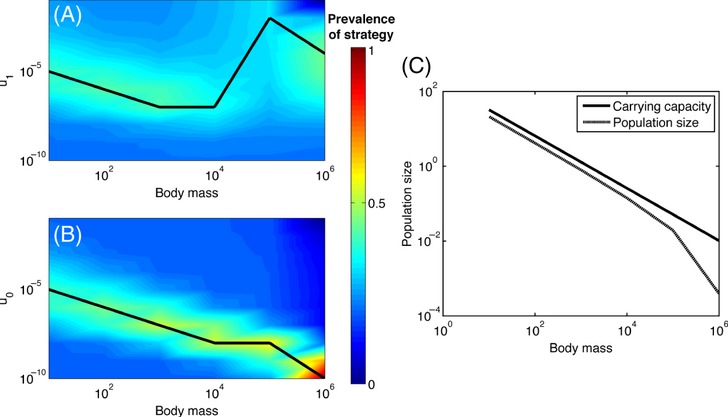
Influence of body mass (in grams) on the prevalence at equilibrium of oncogene activation (*u*_0_, A) and tumor suppressor gene inactivation strategies (*u*_1_, B). The black lines represent the most prevalent strategy. (C) Corresponding population size *N* and the initial carrying capacity *K*. Parameters are identical to Fig. 1, with ρ = 10 and m = 0.002.

We first analyzed how evolution may select mechanisms that decrease the frequency of TSG inactivation or oncogene activation between species, along a gradient of body mass (see Supporting information for sensitivity of this analysis on parameter *g*). We found that an increase in body mass (and consequently cell population size *N*_*c*_) acts in contrasting ways on TSG inactivation (Fig. 2A) and oncogene activation (Fig. 2B).

The rate of oncogene activation decreased gradually with increasing body mass, whereas the rate of TSG inactivation followed an identical pattern until a given threshold, at which point it increased suddenly before decreasing again for larger body masses (Fig. 2B). At this threshold, TSG inactivation and oncogene activation rates evolved toward low values, implying a large decrease in birth rate through the assumed trade-off defined by function *ω*(*u*_0_*,u*_1_) (see section Materials and Method).

The double-hit nature of TSG inactivation can explain why this inflexion point appears only for TSG inactivation rate (*u*_1_). As organisms increase in mass, high rates of TSG inactivation are more tolerated than high rates of oncogene activation as it generates less death from cancer, through its recessive nature, despite a similar reduction of birth rate.

Finally, beyond this body mass threshold, the cost associated with cancer mortality continues to increase when birth is at its maximum. Consequently, the rate of TSG inactivation again decreases, leading to a population size that moves away from its natural carrying capacity (Fig. 2C).

### Profiles of cancer dynamics in natural animal populations

**Figure 3:**
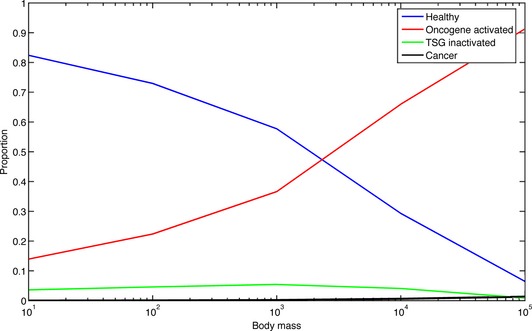
Prevalence at the dynamical equilibrium of individuals with only oncogene activated (*O*), only tumor suppressor gene (TSG) inactivated (*T*) and both oncogene activated and TSG inactivated (C) as a function of body mass (in grams).

We analyzed the expected frequency of individuals with the TSG inactivated, the oncogene activated, or both, for populations spanning a range of body masses. We observed that the proportion of individuals with an activated oncogene at equilibrium increased continuously along the gradient of body mass, up to fixation for the largest organisms (Fig. 3). Individuals with only TSG inactivated are relatively rare, as well as those with both alterations in populations of all masses. It leads to the expectation that most individuals in a population should have an activated oncogene, but only a tiny fraction will have full-blown cancer.

### Experiments to track the selection of protection mechanisms against cancer

Finally, we investigated model scenarios that could be tested experimentally by relaxing the assumption that healthy, oncogene-inactivated, and TSG-inactivated individuals die at the same rate (*d*_*H*_ = *d*_*T*_ = *d*_*O*_). We explored what happens when oncogene activation has immediate and negative consequences on individual fitness with the aim of understanding how protection mechanisms against oncogene activation could arise.

**Figure 4:**
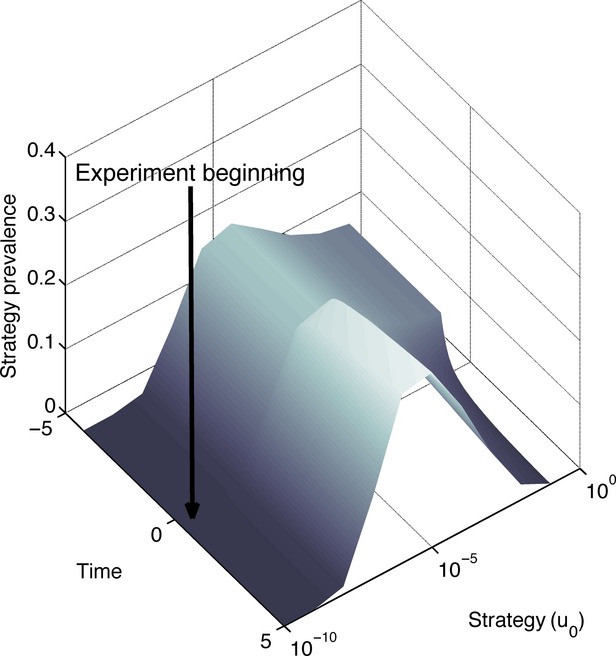
Theoretical results of a potential experiment to track mechanisms for arresting oncogene activation by applying an excessive cost (e.g., through a sacrifice) of individuals with only oncogene activated starting at *T* = 0. Body mass is assumed to be 10 g. Other parameters are the same as previously.

First, we considered an organism with a low body mass (*M* = 10 grams), such as a small rodent. We simulated an experiment lasting 10 generations in which individuals with an activated oncogene have a greater probability of death (parameter *d*_*O*_ = ρ*d*). As expected, Fig. 4 shows that phenotypes with slower oncogene activation, *u*_0_, are favoured in this situation. While frequencies of these oncogenic statuses through time highlight that many different strategies can coexist, we show that the frequency of individuals with slower oncogene activation rate *u*_0_ increase after several generations into the experiment. This gives insight into the identification of mechanisms that could be used to reduce oncogene activation. However, this selection against oncogene activation is also associated with a moderate increase in TSG inactivation (shown in Supporting information). Future research exploring the range of possible proximal mechanisms to reduce oncogene activation needs to consider the possibility of potentially unwanted interactions with TSG inactivation.

## Discussion

In this study, we analyzed from a theoretical perspective the evolutionary dynamics of TSG inactivation and oncogene activation in natural animal populations in relation to body mass. We modeled the evolution of the number and activation rates of TSG and oncogenes in species of increasing size. Our model predicts that the rate of proto-oncogene activation, which depends on the number of proto-oncogenes, becomes low in large species. Because we did not allow the number or rate of TSG inactivation or oncogene activation to drop below a minimum threshold (the maximum mutation rates for activation/inactivation are set at 10^−1^), we observed an increase in the importance of TSG as a cancer suppression strategy in larger species. Consistent with the relative difficultly of obtaining two hits for inactivation of TSGs as opposed to one for activation of proto-oncogenes, the frequency of individuals within populations with inactivated TSG in our simulations was low and remained relatively constant as body size increases. We showed that mechanisms that reduced TSG inactivation vary between species with low and high body mass; a size threshold exists, above which the cost of maintaining low numbers of TSG or proto-oncogenes is unsustainable. In species with large body mass, we showed that oncogenes should be activated in most of the population. Finally, we demonstrated experimental conditions in which mechanisms that reduce oncogene activation may arise rapidly.

We made a number of assumptions to keep our model tractable. First, we used a mathematical derivation for TSG inactivation rate (Nowak et al. 2004) that assumes a large population size. However, if we instead use the relationship associated with low cell population size, as also described by Nowak and collaborators, our conclusions should not change, as this only implies quantitative changes on parameter *σ*_*T*_. Second, we assumed that rates for activation of the proto-oncogene and inactivation of the first allele of TSG, *u*_0_ and *u*_1_, are independent of one another. This assumption affects our conclusions only if, *in fine*, activation of the proto-oncogene, *u*_0_, occurs less often than the product of inactivating the first and second TSG alleles *u*_1_ and *u*_2_, an unlikely situation with, to our knowledge, no known empirical support.

Another assumption made concerns the trade-off specified between oncogene activation rate (*u*_0_), TSG inactivation rate (*u*_1_), and the multiplicative coefficient of birth rate (*ω*). We argue that this trade-off is reasonable, especially when considering the energetic limitations between investment in preventing or repairing mutations and reproduction (Moses and Brown 2003), and has been already assumed elsewhere in different context like evolution of resistance and tolerance to infection for instance (Restif and Koella 2004). This said the shape of this trade-off, especially through the parameter *g*, contributes significantly to the validity of our results. As shown in the Supporting information, we observe that lower values of *g* lead to greater birth rates, which in turn increase oncogene activation and TSG inactivation rates. Conversely, greater values of *g* lead to reduced birth rates and thus to stronger selection on slower oncogene activation and TSG inactivation rates. Then, we suggest that the threshold encountered within our study would require that


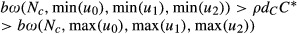


where C^*^ is the population of people having both oncogene activated and TSG inactivated at equilibrium. In other words, the condition for the threshold observed is that the sum of deaths resulting from natural means and cancer remains between the maximal and minimal birth rates. Other shapes of this trade-off could also be considered, such as asymmetric costs between TSG inactivation and oncogene activation, with the conditions cited above remaining valid.

For model simplicity, we assumed that somatic mutation rates are constant through life, regardless of the species and its longevity. Further, we assumed that selective pressures are constant through the course of life, although it is known that fluctuating selective pressures can result from numerous mechanisms, for example*,* accumulation of mutations through longer exposure to mutagens, reduced predator-avoidance behavior with age, etc. An intuitive next step would be to consider some of these through a complete age-structure model to understand the effects on oncogene activation and/or TSG inactivation rates.

We also assume that a clear relationship exists between an organism's mass and the number of evolving cells. In our model, all cells within a body are considered to be candidates for acquiring TSG inactivation or oncogene activation. This is a simplification because not all cells within an individual are capable of self-renewing (Pepper et al. 2007, 2009; Greaves 2010; Sprouffske et al. 2011), the number of evolving cells are likely smaller, which reduces the rate of TSG inactivation and oncogene activation. Furthermore, we assumed that the proportion of cells that are prone to carcinogenesis is similar across the range of body masses (Caulin and Maley 2011).

Previous theoretical works at the population level have addressed similar questions (Nunney 1999; Nunney, 2003). However, these studies focused exclusively on TSG inactivation and that evolutionary dynamics is governed by a balance between mutation and selection rather than by a trade-off, through the function *ω*(*u*_0_*,u*_1_), as in our study. These previous findings are consistent with ours, as the same prediction of a slower TSG inactivation rate is made. In our study, however, we showed that this prediction should be nonlinear because we integrated the fact that energetic limitations also occur between oncogene activation and reproduction. We also reported that oncogene activation and TSG inactivation rates interact with each other by sharing an identical cost, namely death of the individual.

Cancer in natural animal populations has rarely been studied, with only a handful of studies from the conservation literature, where detecting cancer in these species may be an indicator of a disturbed environment (McAloose and Newton 2009). The problem of Peto's paradox suggests that more attention should be placed on detecting cancer in natural animal populations with different masses (Peto et al. 1975; Caulin and Maley 2011; Roche et al. 2012), with the goal of being able to identify genetic mechanisms that some species use to protect against cancer. While current attempts to address this paradox focus on general mechanisms of tumor formation (Nagy et al. 2007) or cataloguing all potential differences between species with small and large body masses (Caulin and Maley 2011), we have adopted here a complementary way of studying cancer in natural animal populations. In this work, we explored the consequences of a simple and instructive view of cancer emergence – the role of TSG inactivation and oncogene activation on the evolution of species. This has allowed us to suppose mechanisms governing rates of oncogene activation and TSG inactivation can be interconnected but do not follow the same pattern. Our model simulations show a trend with a small increase in cancer rates in individuals of increasing size consistent with cancer prevalence observed for Peto's paradox. Thus, once possible solution to Peto's paradox predicted from our model is that large species may reduce the number of oncogenes (or the ease of their activation).

One of the primary goals of our study was to show how we could identify key species to study protection mechanisms against cancer. As previously said, we found a threshold in the gradient of body mass, when population size moves away from theoretical carrying capacity. Estimating theoretical carrying capacity of a wide spectrum of species, through environmental niche modeling for instance, and comparing them with the real carrying capacities could highlight the identity of these species at this threshold. These could then be used as a starting point to disentangle the mechanisms slowing down TSG inactivation or oncogene activation. We also suggest that experiments focusing on how natural selection against oncogene activation might arise. Identifying such mechanisms in laboratory models could be especially insightful in cancer prevention (Gatenby and Maini 2003), as well as open new opportunities for cancer therapy development.

Because large organisms such as whales should have evolved multiple mechanisms slowing down TSG inactivation and oncogene activation (Roche et al. 2012), disentangling the role of each mechanism is especially tricky. Furthermore, comparing such mechanisms between relevant pairs of organisms with one order of magnitude difference in their body masses should provide additional insights, suggesting that sequencing the whole genome of intriguing species like the naked mole rat that exhibits roughly no cancer (Kim et al. 2011) would be even more useful when its genome can be compared with the genomes of a relevant set of species.

## Supporting Information

**Figure S1 and S2:** Sensitivity analysis of figure 4 with different values of parameter g.

**Figure S3.** Evolutionary dynamics of TSG inactivation rate generated by simulation shown in figure 4.
