# Supplementary material for: Peto's paradox revisited: theoretical evolutionary dynamics of cancer in wild populations
Source: Evol Appl. 2012 Nov 22;6(1):109–16. doi: 10.1111/eva.12025 (PMC3567476; doi:10.1111/eva.12025)
Supplement: Supplementary file 1 [file eva0006-0109-SD1.docx]

**Peto’s paradox revisited: Theoretical evolutionary dynamics of cancer in wild populations**

**Supplementary materials**

***Two-dimensional model with mutation process :***


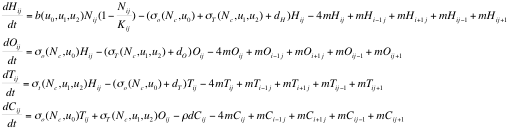
***Sensitivity to the trade-off values through the case of experimentation***

In order to assess the generality of our results, we address different values of the parameter *g* that shapes the trade-off between birth rate, *u_0_* and *u_1_*. We examine the case shown in figure 4 (M=10, d_O_=10d_H_ when T>0) with different values of this parameter *g* (figure S1 and S2).

*
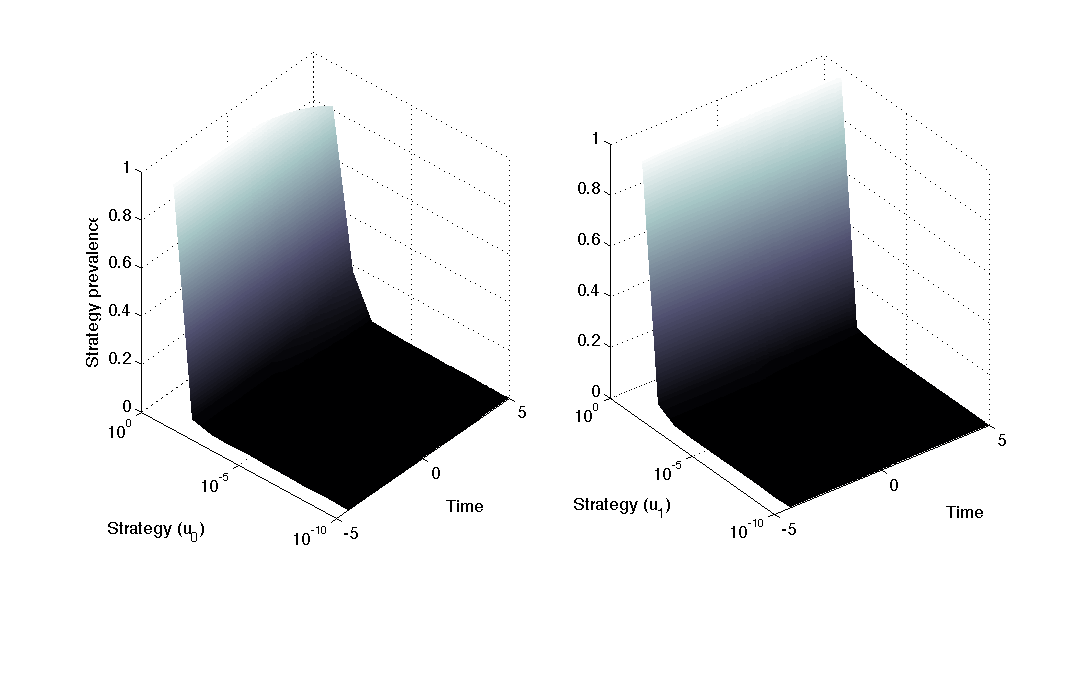
*

**Figure S1: Same simulation than figure 4, but with g=2.10^-2^.**

*
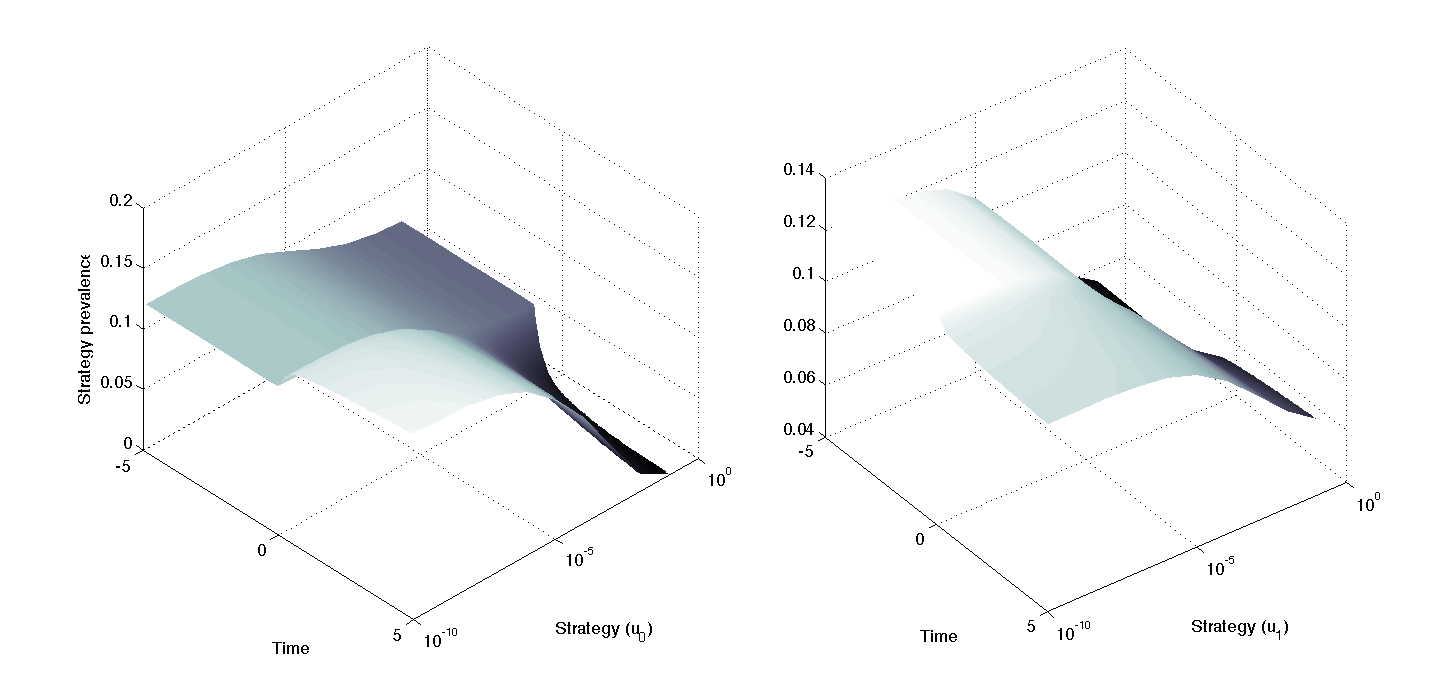
*

**Figure S1: Same simulation than figure 4, but with g=2.10^-16^.**

As said in the main text, we observe that a lower value of *g*, leading to greater values in birth rates, allows sustaining high level of oncogene activation and TSG inactivation rates. On the opposite, greater value of *g* leads to a reduced birth rate and then to a stronger selection of slower oncogene activation and TSG inactivation rates. Then, our results remain valid when the addition of natural mortality with mortality from cancer does not exceed and goes under the maximal and minimal birth rate respectively.***TSG inactivation of experimentation shown in the main text***

We show here the experiment proposed in the main text, and displayed in figure 4, has moderate consequences TSG inactivation rates (figure S3)*.
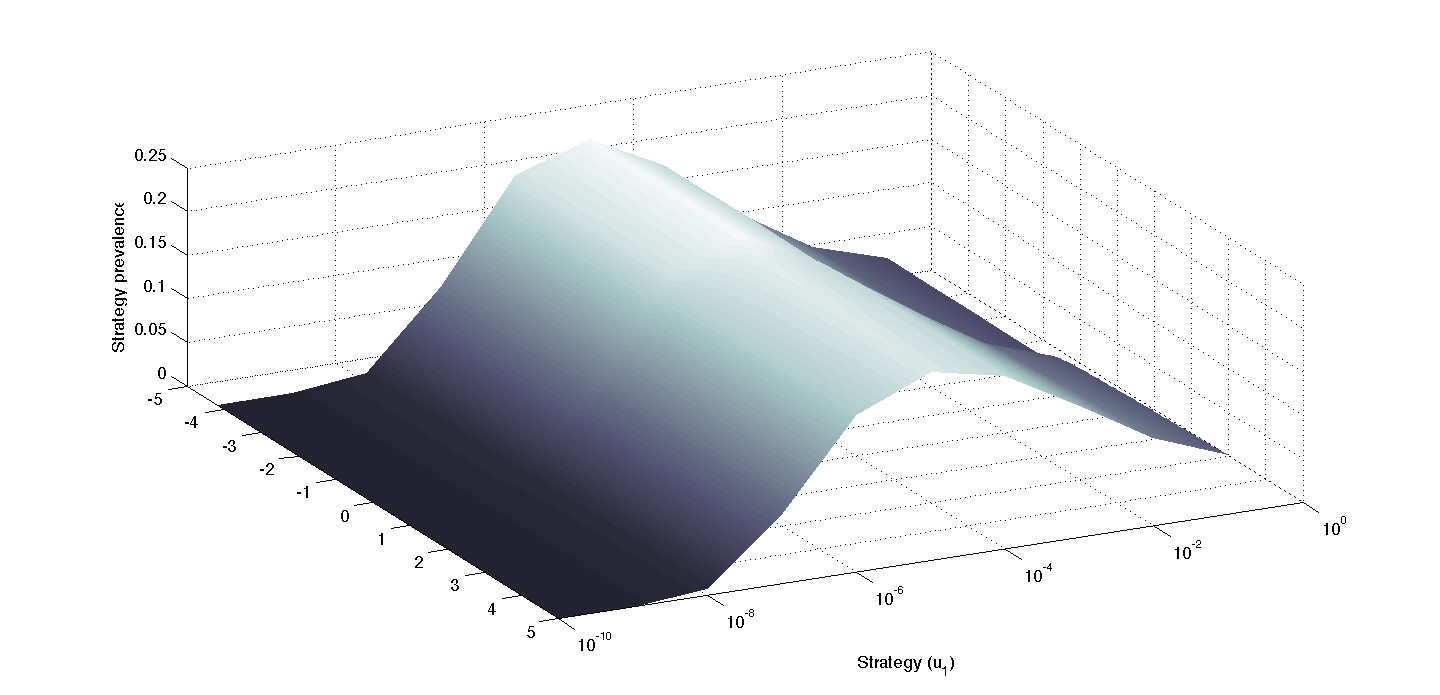
* **Figure S3: Evolutionary dynamics of TSG inactivation rate resulting from experiment shown in figure 4.** **Experiment starts at T=0.**
